# The Decision and Discussion of the Liverpool Meeting

**Published:** 1912-07-27

**Authors:** 


### THE DOCTORS AND THE ACT.

### The Decision and Discussion of the Liverpool Meeting.

The representative meeting of the British Medical Association opened at Liverpool on Friday last week. The circumstances were of exceptional interest, as the delegates met chiefly to decide the attitude of the medical profession to national insurance, and consequently its policy as regards the National Insurance Act. The day before the meeting opened Mr. Masterman stated that the decision arrived at by the meeting would be accepted as final by the Government. About 270 representatives were present. At the last meeting of the representative body, which was a special meeting held at the Guildhall, London, in February, certain recommendations in regard to insurance were decided. A State Sickness Insurance Committee was appointed, with power to report to the Council, and from the Council to the divisions, but with no power to conclude negotiations without referring to the representative body. It is the report, which has now been circulated, that has had to be decided upon, and it contains two sets of alternative recommendations which have been made by the Council :—

I.—To break off negotiations :—

(1) That the Government be informed that the Association adheres to its minimum demands as formulated in the letter of February 29, 1912, and since elaborated in interviews with the Chancellor of the Exchequer; (2) that the Association calls upon all members of the Association who are members of Advisory Committees in connection with the National Insurance Act, and also on other medical members of those committees who are in sympathy with the policy of the Association, to withdraw from these bodies; (3) that the British Medical Association calls on all those practitioners who have signed the undertaking, and on all other practitioners who desire the unity of the profession, to refrain from applying for or accepting any post or office of any kind in connection with the National Insurance Act until such time as the Government has satisfied the Association that its demands will be met.

II.—To continue negotiations :—

That a State Sickness Insurance Committee be appointed, with power to negotiate with the Commissioners and to ascertain to what extent the demands of the Association are likely to be met; to consider and report on the regulations of the Commissioners as soon as available; and to report on the whole situation as soon as possible to a special representative meeting; and that meanwhile all steps be taken to perfect the organisation of the profession and to increase the Central Insurance Defence Fund.

On July 19 the annual representative meeting of the Association was opened at St. George's Hall, with Dr. E. J. Maclean, of Cardiff, in the chair. Sir James Barr, the president-elect, welcomed the delegates, and it was decided to postpone consideration of the insurance question till Saturday. Financial questions were then dealt with, and the Council was directed to prepare a report on the desirability of increasing the subscriptions temporarily or otherwise. In the discussion, which led to this result, the large expenditure of the Association incurred by its action in defending the interests of the profession with respect to the Insurance Act was considered, and the proposal temporarily to increase the subscriptions was warmly welcomed by several Colonial members, who, while unable to pledge their oversea colleagues, repudiated a previous suggestion that it would be unfair to increase the subscriptions of practitioners who were resident abroad. A proposal to seek the opinions of the divisions as to the desirability of converting the Association into a body registered as a trade union marked an animated discussion on both sides, and finally a motion was carried to postpone its further consideration for twelve months.

Intense interest was excited in the meeting on Saturday, which was watched by many, including ladies, who were not members of the representative body. The sitting was held in the small concert hall which adjoins St. George's Hall. Officials and delegates to the number of 270 crowded the hall, any over-space in which was fully occupied by spectators. The meeting quickly resolved itself into committee on the Insurance Act, and the proposal to appoint Mr. T. Jenner Verrall chairman of the committee having been disallowed on the ground that he was chairman of the State Sickness Insurance Committee, Dr. Buist, deputy chairman of the representative meeting, was appointed. The meeting resolved to consider in committee the reports presented to it by the Council, including that embodying the reply of the Insurance Commissioners on June 25 to the letter addressed to them by the Association on February 29, and the observations of the State Sickness Insurance Committee thereon. This report has already been published. The meeting resolved that the proceedings in the committee stage should be conducted *in camera*, and that the press should not be admitted.

### Mr. Jenner Verrall's Speech.

On going into committee, Mr. T. Jenner Verrall, speaking as chairman of the State Sickness Insurance Committee, in the course of his speech said : During the intervening months an effort has been made to retrieve a position which had been unsatisfactory from the first, inasmuch as the medical profession had not been consulted at an early stage. I have regretfully to admit that the result of the committee's strenuous work is correctly summed up in the opinion of the Council attached to the second part of the report to the effect "that no material concession had been made by the Chancellor of the Exchequer since February last." The State Sickness Insurance Committee, over which I presided, is about to die a natural death, and if the meeting desires to place an epitaph over their body I would suggest that it should be the words : "They extracted an answer from the Commissioners." The report of the committee consists of three parts. The first merely relates the work which the committee has done, and sets out the various communications it has addressed to the Commissioners. The second part contains the reply eventually received from the Commissioners with the observations of the committee. The reply has been received after two interviews with the Chancellor and the Commissioners. At these interviews the committee has been asked to support by evidence the demands of the profession, and the committee has accordingly sent in a document, already published, in support of the demands of the profession, which, in my opinion, including the monetary demands, did not exceed in any particular what it had a right to ask.

At the first of the two interviews with the Chancellor of the Exchequer he put before them certain considerations and conclusions based on certain assumptions which proved to his satisfaction that the average income which a member of the medical profession would receive would run into four figures, but neither at that interview nor at the second interview was any criticism of the memorandum sent in by the Association attempted, beyond a question raised by the Chancellor as to the number of assistants, as to which the Chancellor was undoubtedly in error. The Chancellor had asserted that the memorandum presented by the Association was based to a very large extent on assumed figures; that was perfectly true, but it was true also that the Chancellor's own calculations were based on guesswork. The Chancellor had asked the assistance of the committee in examining the accounts of all practitioners in certain towns, and the committee had consented not to oppose the Chancellor's proposal, after ascertaining the exact questions which were to be put and the towns in which the inquiry was to be made, reserving to the Association, and to every member of the profession, the right to criticise the results when they were obtained.

### Sir W. Plender's Report.

He did not dispute that Sir William Plender's report was a perfectly valid document, so far as the figures it contained were concerned, but the whole value of the report depended upon what could be deduced from it. Arguments had been based upon it which did not hold water. The members of the profession in the towns selected deserved thanks for the readiness with which they consented to the inquiry being made, and their action showed that the medical profession wished to place no stumbling-block in the way of the Government arriving at a just decision.

The meeting, which began at 9.30 a.m., continued in committee until the adjournment at 8 p.m.

### The Alternative Policies.

The whole of this time was spent in considering the two alternative policies which have been outlined above. The Chairman had the names of eighty speakers sent up to him. The large majority of these were in favour of breaking off negotiations. Among the speakers were Dr. Beaton, Dr. Dryland, Dr. Easterbrook, Dr. J. R. Fuller, Dr. Major Greenwood, Dr. R. W. Henry, Dr. J. A. Macdonald, Dr. M'Kenzie Johnston, Mr. Meade, Dr. Metcalfe, Dr. O'Sullivan, Dr. Pope, Mr. Raiment, Dr. W. Johnson Smyth, Mr. Sturge, and Mr. E. Tredinnick. Speakers who were against the unconditional rupture of negotiations were Dr. Durrant, Dr. Keay, and Dr. Maclean. Criticism was levelled against such recent events as the Chancellor's letter, Sir William Plender's report, and Mr. Masterman's pronouncement on the eve of the Liverpool meeting.

### Mr. E. B. Turner's Speech.

After luncheon, to a still crowded house Mr. E. B. Turner, of the Kensington division, made a trenchant speech, in which he deprecated any further expenditure of time in negotiations, which, he said, could have no satisfactory issue. He recalled the fact that the representative meeting in February had instructed the Council to inform the Commissioners in plain and unmistakable language that unless the minimum demands of the Association were conceded the Association intended to call on its members to decline to work the Act. These demands, he proceeded, had not been conceded, and the Association would stultify itself if it retreated now from the position which it had deliberately taken up. At the end of the five minutes allotted to him, the favour with which his remarks were regarded by his audience was so marked that in deference to the general wish additional time was allowed to him. When Mr. Turner had concluded he received an ovation, and his speech was decidedly the most remarkable feature of the discussion.

### Sanatorium Benefit.

At the sitting in committee the third recommendation, which had been under discussion on Saturday, was carried, when it had been amended to exclude sanatorium benefit, by a majority of more than two to one, sixteen delegates abstaining from voting. Then followed a debate on numerous resolutions for safeguarding the general practitioner under the Act in cases where the patients are receiving sanatorium benefit.

On Tuesday afternoon and evening Sir James Barr, the president-elect, took the chair on its vacation by Professor Saundby, the retiring president. In the morning the representative body continued to meet in committee, and the question of admitting accredited reporters was again discussed, the consideration of the Act continuing in semi-privacy. Though the resolution calling on doctors to refuse to work the Act was amended on Monday, so as to exclude sanatorium benefit, the question was again debated on the report stage. Whatever is decided by the representatives at the report stage is binding upon the Association. An amendment to refer back the question of sanatorium benefit to the divisions was defeated by a two-thirds majority. On Tuesday afternoon the report stage of the deliberations on the insurance question was reached, which had occupied the committee from Saturday till Tuesday midday. An official announcement contained the following decision and resolution:—" That the Government be informed that the Association adheres to its minimum demands as formulated in the letter of February 29, 1912, and since elaborated in interviews with the Chancellor of the Exchequer."

The meeting also adopted the following resolutions:—

" That the Association calls upon all members of the Association who are members of Advistory Committees in connection with the National Insurance Act, and also on other medical members of those committees who are in sympathy with the policy of the Association, to withdraw from these bodies."

" That all medical practitioners who have accepted office on any of the provisional Insurance Committees throughout the country be called upon to resign their positions."

It was provided that these resolutions should not apply to Ireland.

### The President's Address.

On taking the chair as president, Sir James Barr said: The general trend of opinion in the profession is veering round to my own view about the Insurance Act, which I consider the most gigantic fraud which has ever been perpetrated on the public since the South Sea Bubble. (Cheers.) The earlier one was soon pricked, but there are so many vested interests being created in connection with this later one that it will be a difficult matter to get rid of it. The only people who would benefit by it, he added, were the officials, who might be as well employed in earning an honest livelihood

Turning to other subjects, the President said he had chanced to read the Huxley Lecture on " Life and Consciousness " by Henri Bergson, who asks, with reference to mankind " What are we? What are we doing here? Whence do we come and whither do we go? " and it occurred to him that these questions might be appropriately asked of the medical profession. During the last century or more they had been very busy trying to find out the causes of disease, the best methods of their elimination, and the proper treatment of our patients. They had to a large extent succeeded; so that a selective death-rate, which was Nature's method of eliminating the unfit, has at least been partially suspended by their efforts. How far the human intellect might yet succeed in unravelling many of the mysteries of life they could not at present say; but he had no doubt that in the process of evolution the human intellect would be carried to a much higher plane than it had at present attained, if, as Mr. Balfour had said, the forces of disintegration did not overcome those of civilisation.

The Evolution of Medicine.

Natural evolution, and the evolution of the art and science of medicine, ran on different lines. Nature was lavish in the production of life and prodigal in its destruction; her efforts were directed to the improvement of the species, she cared nothing for the individuals except to adapt them to the environment. This spelt elimination of the unfit. Medicine, on the other hand, had been evolved for the benefit of individuals. They had successfully interfered with the selective death-rate which Nature employed in eliminating the unfit; but they had made no serious attempt to establish a selective birth-rate. The same maudlin sentimentality which often pervaded the public not infrequently affected the medical profession. They had often joined forces with self-constituted moralists in denouncing the falling birth-rate, and had called out for quantity, regardless of quality.

They had often assumed the position of guides, philosophers, and friends, but in the main they were individuals who dealt with the prevention and treatment of disease. That was what they would continue to do if some of their ignorant legislators, who knew nothing, and cared less, about physiological laws, had their way. They must instil into the public mind a recognition of the value of intellectual and physical health. If this achievement was to be accomplished they must begin with the unborn. Above all, they must breed for intelligence. Of course, they could not make all equal—there was no such thing as equality in Nature, and never could or would be, but they could raise the average plane and get rid of the present decadence. The time was now ripe for dealing with mental weaklings, and they would be dealt with effectually in all progressive nations. The advanced thought of the intelligent would demand something more than treatment from the medical profession. That would place physiology on a higher plane than pathology, and would require a more careful study of the laws of health than of the tendencies to degradation and disease. Such teaching was bound to come, and advanced physiology and biology must lead the way. The hardworked wage-earner had no fair chance, as he had not only to support his own progeny, but was taxed almost out of existence to maintain the extravagance of wasteful Governments and of a sympathetic irrational public. "The right to live" was not a doctrine held by any political economist, but one that had been forced upon them by the evolution of sympathy. They had no moral right to leave a legacy of mental obliquity and of physical decadence to weigh upon the next generation. He expressed his regret that this necessity for the higher teaching of physiology was as yet very inadequately recognised, even by the General Medical Council. As had long been advocated by the British Medical Association, they must have a properly regulated State examination, and the vested interests of corporations must not be suffered to prevail against the public weal. Mere examining corporations which had done practically nothing for medical education, and were chiefly engaged in extracting fees from students, should be allowed to retire into obscurity. There was then plenty of good work for an able statesman—a man determined to lead and to prevent the present policy of drift. They must recognise that the outlook of the profession, though at present somewhat dismal, was merely a transition stage which would soon pass away.

The President-Elect, Dr. Hollis, who will preside over the Brighton meeting next year, was introduced to the delegates.

It was decided to establish a fighting fund to resist those parts of the Insurance Act which adversely affect the profession, the minimum subscription to the fund being fixed at £20.

